# Digital Contact Tracing Against COVID-19 in Europe: Current Features and Ongoing Developments

**DOI:** 10.3389/fdgth.2021.660823

**Published:** 2021-06-17

**Authors:** Alessandro Blasimme, Agata Ferretti, Effy Vayena

**Affiliations:** Health Ethics and Policy Lab, Department of Health Sciences and Technology, ETH Zürich, Zürich, Switzerland

**Keywords:** APP, digital contact tracing, COVID-19, governance, privacy, epidemiology, Europe

## Abstract

The SARS-CoV-2 pandemic is a public health challenge of unprecedented scale. In the midst of the first wave of the pandemic, governments worldwide introduced digital contact tracing systems as part of a strategy to contain the spread of the virus. In Europe, after intense discussion about privacy-related risks involving policymakers, technology experts, information technology companies, and—albeit to a limited extent—the public at large, technical protocols were created to support the development of privacy-compatible proximity tracing apps. However, as the second wave of SARS-CoV-2 sweeps the continent, digital contact tracing in Europe is evolving in terms of both technological and governance features. To enable policymakers to harness the full potential of digital health tools against SARS-CoV-2, this paper examines the evolution of digital contact tracing in eight European countries. Our study highlights that while privacy and data protection are at the core of contact tracing apps in Europe, countries differ in their technical protocols, and in their capacity to utilize collected data beyond proximity tracing alone. In particular, the most recently released apps tend to offer users more granular information about risk in specific locations, and to collect data about user whereabouts, in order to enhance retrospective contact tracing capacity. These developments signal a shift from a strict interpretation of data minimization and purpose limitation toward a more expansive approach to digital contact tracing in Europe, calling for careful scrutiny and appropriate oversight.

## Introduction

The SARS-CoV-2 pandemic is a public health challenge of unprecedented scale. Worldwide, 163 million people have tested positive for SARS-CoV-2 and 3.38 million have lost their life to the coronavirus disease (COVID-19) (1). As of May 2021, Europe alone has had more than 31 million cases and 700 thousand deaths according to the most recent estimates of the European Center for Disease Control and Prevention (2). Since late summer 2020, Europe has faced a resurgence of new cases as a second wave of SARS-CoV-2 spread across the continent, placing health systems under severe pressure and forcing governments to reinstate restrictions similar to those adopted in the first quarter of the year.

Alongside restrictions to population movement during the first wave of the pandemic, governments throughout the world introduced digital contact tracing (DCT) systems, in the hope that this new digital health technology would help contain the spread of the virus (3). DCT systems mostly come in the form of smartphone apps that, using technologies commonly present in such devices–such as the Bluetooth data exchange standard or the global positioning system (GPS)–can keep track of the proximity between devices that have the app installed. Proximity data can be used to infer the risk that two users might have been close enough and for a sufficient amount of time to infect each other with the SARS-Cov-2 virus. Once a user tests positive for the virus, DCT apps can send an alert to other users who have been in close contact with her according to the proximity data recorded by the system. Alerted users can then test and isolate thus reducing the circulation of the virus in a given population. Asian countries were among the first to adopt DCT. Recognizing the public health potential of DCT, many European countries followed suit during the spring, developing national DCT systems in an attempt to expand their contact tracing capability.

Despite the promising potential of DCT, its introduction gave rise to intense debate over ethical, legal, and societal implications (ELSI). In particular, some characteristics of the Asian approach (mandatory use, centralized protocols, GPS- or cell tower-based geolocation) are seen by many as incompatible with European legal provisions and ethical views about the value of individual privacy.

For this reason, European policymakers, in close collaboration with technology experts and IT companies, started developing DCT standards based on the exchange of anonymized Bluetooth data. The European approach to DCT is defined in specific guidelines issued by the European Commission (EC) on April 17, 2020. This guidance is centered around the principle of data minimization, including precisely defined limits for data disclosure, use, and storage (4).

Meanwhile, in mid-April, the eHealth Network (comprising representatives of authorities responsible for digital health in the 27 EU Member States plus Norway) published a common toolbox, specifying essential requirements for European DCT apps. This toolbox emphasized a preference for decentralized protocols which store anonymized proximity data exclusively on users' mobile phones, over protocols storing data on centralized servers that are run by national health authorities. In particular, echoing the opinion of the European General Data Protection Board, this guidance underscored decentralized approaches as better suited to “keep personal data processing to the absolute minimum,” enhance citizens' willingness to download and use DCT apps, and prevent “risks of data breaches and cyberattacks” (5).

At this time, many European technology experts were still collaborating on a centralized protocol called the Pan-European Privacy-Preserving Proximity Tracing protocol (PEPP-PT). Ultimately, though, some members of the PEPP-PT project resigned from this consortium in order to form a new protocol (6). The privacy-preserving decentralized protocol (Decentralized Privacy-Preserving Proximity Tracing, or DP-3T for short) was developed by a number of European academic institutions, in conjunction with the Swiss Federal Institutes of Technology (ETH Zurich and the EPFL of Lausanne).

In the meantime, Google and Apple released an application programming interface (API) to implement this protocol on Apple and Android mobile operating systems (Google/Apple Exposure Notification system, or GAEN for short). Most decentralized DCT systems in Europe, including the Swiss model, run on this protocol. Countries such as Germany and the UK used the centralized model initially, but adopted the decentralized scheme powered by Google and Apple for the final version of their national DCT apps, introduced on June 16 and September 24, 2020, respectively (7, 8).

At the time of writing, 19 of the 27 EU Member States plus Switzerland have created a national DCT app (9). Of these, only France and Hungary have opted for a centralized solution (10).

In this comparative study of national proximity tracing apps, we seek to characterize the European approach to DCT, and to examine its evolution between the first and second waves of SARS-CoV-2. Our analysis shows that European DCT systems, to some extent, are evolving to incorporate new features extending their capabilities beyond mere proximity tracing—a development that calls for careful scrutiny and adequate oversight.

## Methods

In order to examine the evolution of the European DCT landscape, we collected information from primary sources about national DCT apps in the following countries: France, Germany, Ireland, Italy, the Netherlands, Switzerland, and the UK (including England, Wales, and Scotland).

We included DCT systems released between March and October 2020. All the systems we included in our analysis revolve around a smartphone app as their key implementation technology. For inclusion in our study, the language of the app had to be English, French, Italian, or German (languages spoken by the authors). For each app, we collected *Privacy Policy* and the *Terms of Use* documentation from the app itself or its associated website. When available, we also analyzed the “FAQ section” and “press release” documentation, which usually contain a series of questions and answers, as well as concise information about the app's functionality and data processing. A list of the primary sources analyzed is available as Supplementary Material. Each source was archived (on archive.org) as it appeared at the time of review.

From this documentation, we first extracted and recorded general information and technical features for each DCT app (via MS Excel). Next, we imported the retrieved documents into Nvivo for qualitative content analysis. Two researchers (AB and AF) inductively created analytic codes from the text until thematic saturation was achieved (11). Semantically similar codes were further grouped into themes and subthemes. Two researchers (AB and AF) coded the text independently and resolved any coding discrepancies through discussion.

For comparative purposes, we collected information from secondary sources about national DCT systems in Asia. A list of these sources is available as Supplementary Material.

## Results

### Common Characteristics

Table 1 provides a summary of select descriptive features for each included DCT app. A certain degree of similarity is evident across the analyzed DCT systems. For example, all of them were developed in public-private partnerships between the state (or national health authority), software development companies, and, at times, research institutions. Furthermore, all of the apps function on a voluntary basis, in order to safeguard individual freedom. Moreover, a strong focus on privacy preservation and data protection is a common feature of the European approach to DCT. However, not all countries use the same architecture to achieve this aim.

**Table 1:** Characteristics of DCT systems in selected European countries.

	**Switzerland**	**Italy**	**Germany**	**Ireland**	**Netherlands**	**United Kingdom**		**France**
						**Scotland**	**England & Wales**	
	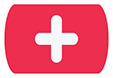	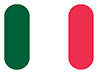	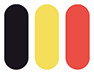	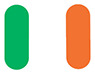	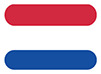	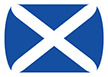	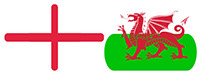	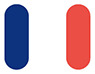
Total COVID-19 cases^§^	426,199	2,038,759	1,640,858	85,394	754,171	2,256,009		2,507,532
Cumulative prevalence per 1 million population^§^	49,245,25	33,719,77	19,584,4	17,293,99	44,013,81	33,232,31		38,415,77
Total deaths^§^	6,508	71,620	29,778	2,200	10,974	70,405		62,197
App name	SwissCovid	Immuni	Corona-Warn-App	COVIDTracker	CoronaMelder	ProtectScotland	NHS Covid-19	TousAntiCovid
Release date	25.05.20	15.06.20	16.06.20	07.07.20	17.08.20	14.09.20	24.09.20	22.10.20
N. of downloads	2,863,858^a^	10,072,742^b^	24,200,000^c^	2,700,000^d^	4,330,264^e^	1,739,806^f^	20,739,925^g^	11,897,809^h^
Developed in public-private partnership	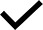	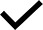	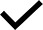	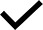	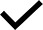	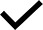	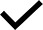	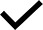
Voluntariness	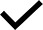	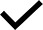	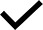	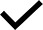	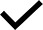	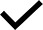	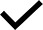	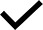
De-centralized protocol	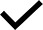	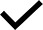	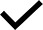	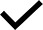	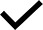	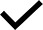	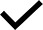	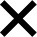
Exposure parameters	1.5 meters for 15 min	<8 meters 10 min	2 meters for 15 min	2 meters for 15 min	“near” for 15 min	2 meters for 15 min	2 meters for 15 min	2 meters for 15 min
Data retention: Random ID Exposure code	14 days 14 days	14 days 14 days	14 days 21 days	14 days 14 days	14 days 21 days	14 days 14 days	14 days 14 days	14 days 14 days

§ *at 28.12.2020 source: https://covid19.who.int*.

a *at 21.12.2020 source: https://www.experimental.bfs.admin.ch/expstat/en/home/innovative-methods/swisscovid-app-monitoring.html*.

b *at 28.12.2020 source: https://www.immuni.italia.it/dashboard.html*.

c *at 17.12.2020 source: https://www.rki.de/DE/Content/InfAZ/N/Neuartiges_Coronavirus/WarnApp/Archiv_Kennzahlen/Kennzahlen_18122020.pdf?__blob=publicationFile*.

d *at 28.12.2020 source: CovidTracker App*.

e *at 23.12.2020 source: https://github.com/minvws/nl-covid19-notification-app-statistics/blob/main/statistics/appstore_statistics.csv*.

f *at 28.12.2020 source: ProtectScotland App*.

g *at 16.12.2020 source: https://www.gov.uk/government/publications/nhs-test-and-trace-england-statistics-10-december-to-16-december*.

h *at 28.12.2020 source: TousAntiCovid App*.

The majority of DCT apps rely on decentralized protocols. These apps operate with the privacy-preserving technology framework released by Google and Apple, which allows matching codes to be kept on the user's phone, and in the case of a positive test, fetches only an anonymized ID from a centralized database, in order to check for high risk contacts. Among the apps we analyzed, only the French *TousAntiCovid* adopts a centralized approach to data storage. To justify this decision, the French government argued that the Google/Apple system contradicts the digital sovereignty of the state and does not provide sufficient privacy safeguards, as sensitive data about positive cases, albeit encrypted, are accessed by users' apps (12–14). Moreover, as the FAQ section of the French app specifies, “*the Government considers that protecting the health of the French people is a mission that is the exclusive responsibility of the State and not of private international actors*”^1^ (15).

From a technical perspective, European DCT apps employ similar exposure parameters (two meters apart for 15 min) to notify app users of a potentially dangerous contact. Taking a precautionary approach, the German *Corona-Warn-App* uses the most stringent exposure parameters, alerting a user who is within eight meters and for at least 10 min from an individual with a confirmed SARS-CoV-2 infection. The French *TousAntiCovid* employs the least stringent criteria of one meter apart for 15 min.

Despite differences in data storage locations across countries, we noted that data retention periods are consistent, both for randomly generated ID codes as well as temporary exposure codes. Randomly generated ID codes are generally stored for 14 days, while positive exposure codes are kept for 14 (Ireland, Italy, France, Netherlands, Scotland, and the UK) or 21 (Germany and Switzerland) days.

All of the reviewed systems collect statistical data concerning the number of users who downloaded the app, the number of apps actually in use, the positive cases uploaded to the system, the number of alerts sent to users, and the functioning of the app (e.g., Bluetooth signal strength, success of the data exchange, and the time at which the data must be destroyed). Some apps such as *SwissCovid* (Switzerland), *Immuni* (Italy), and *Corona-Warn-App* (Germany) have dedicated web pages offering aggregate information on how the respective DCT systems are being used (16–18). The apps also collect metrics data for public health surveillance, such as the day, time, and duration of a contact; whether the infected user is asymptomatic; the 1st day of illness; and the date of testing. Countries may retain such anonymous data for epidemiological surveillance or research purposes, however retention periods vary across countries. In Italy, metric (i.e., aggregated statistical) data is kept until the end of the emergency, but no later than 31 December 2021 (a limit previously set to the end of 2020.) In Ireland, England, and Scotland, metric data are retained, respectively for at least 7 years, 20 years, and indefinitely.

The seamless functioning of national DCT apps across borders motivated the European Commission to create an EU-wide system called *getaway*, to enable interoperability and help break the chain of COVID-19 infection across borders. The *getaway* would allow users who have installed one DCT app to travel to another participating European country and still receive contact tracing alerts (18). So far, however countries with interoperable apps include only Croatia, Denmark, Italy, Ireland, Germany, Latvia, the Netherlands, Poland, and Spain.

### Country-Specific Features

European DCT apps differ in three respects: what happens upon notification of a contact with a positive case; how positive test results are handled; and additional features beyond proximity tracing.

In all cases analyzed, DCT apps advise users on what to do upon notification of close contact with someone who has tested positive for SARS-CoV-2. Most apps give users instructions for how to self-isolate, register for testing, and contact health authorities if symptoms emerge. The Irish *COVID Tracker* app allows users to voluntarily add a phone number, which is shared with health authorities. In case of close contact, the user not only is alerted by the app, but also phoned by the health authority that provides information about next steps and eventually arrange a COVID-19 test.

Each country follows its own procedure for uploading a positive test result into the DCT system. In Scotland for instance, health authorities send an exposure code via SMS to users who tests positive. Users enter the code, active for 72 h, into the *ProtectScotland* app. In France, the code is sent to users in a link via email, and via post as a QR code. Users must thus enter personal information (mobile number, email, address) in order to communicate the outcome of a positive test and trigger notification to other users. Other countries have chosen methods which avoid this requirement. Users of the German *Corona-Warn-App* can scan a QR code linked to test results, automatically activating the exposure code. In Switzerland, Italy, and the Netherlands, users must phone the health authority upon notification of a positive test result, in order to activate the exposure code.

**Figure 1:**
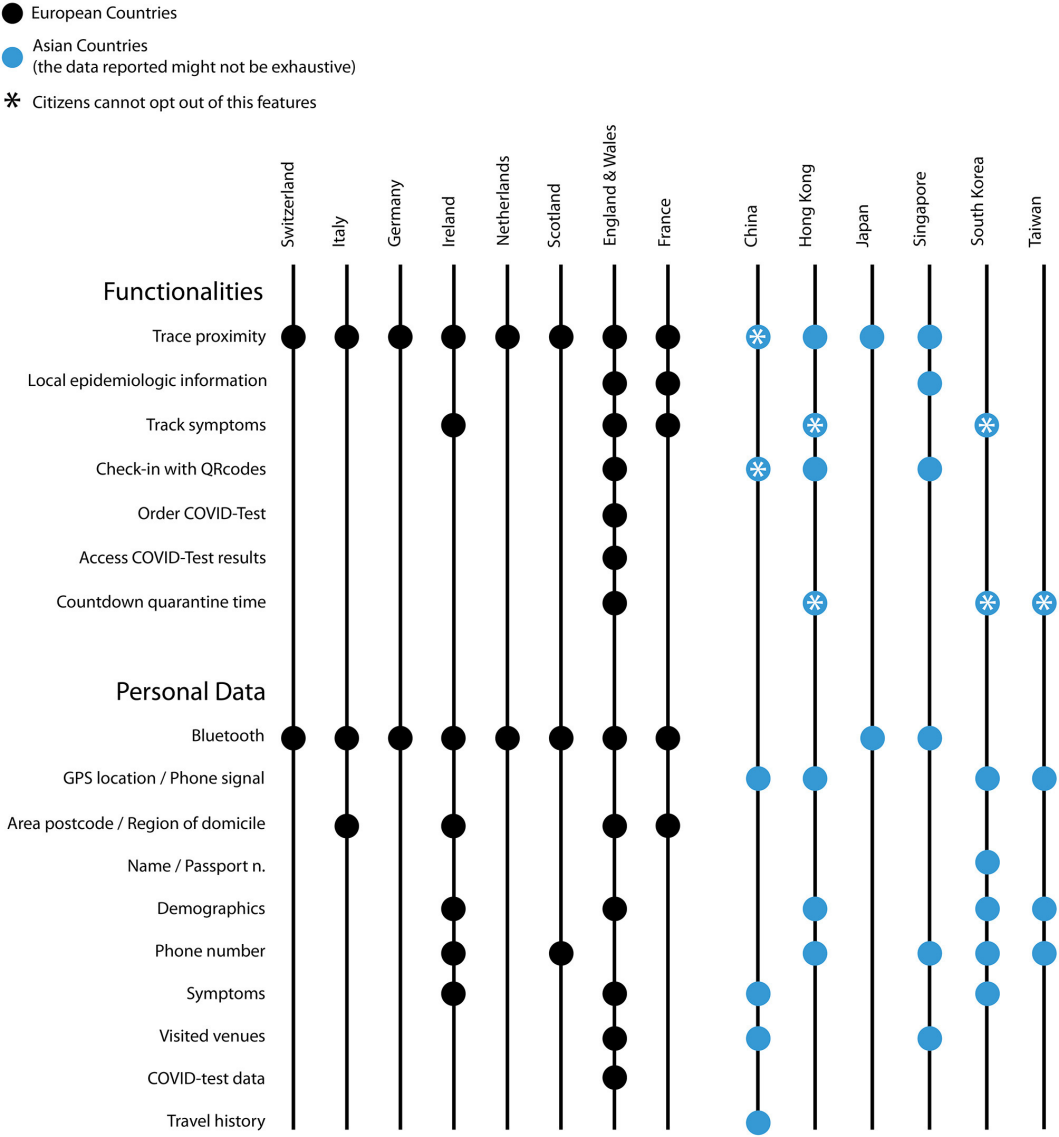
Features of DCT systems in selected European and Asian countries: functionalities and types of personal data collected. (Updated to 31.10.2020).

Our qualitative assessment explored the evolution of DCT apps as one component of broader policy efforts intended to curb the economic and public health effects of the pandemic. The most recently released DCT apps were introduced after the summer, when a second wave of SARS-CoV-2 was already apparent in most European countries. At this time, some national apps released features that went beyond simple proximity tracing (see Figure 1). For instance, the French *TousAntiCovid* [the successor to a previous app called *StopCovid*, which was downloaded by a mere 2.6 million people and therefore replace by *TousAntiCovi*d (19)] expanded its functionality, allowing users to enter their postal code to receive more granular information about the local epidemiological situation. Moreover, users of the French app can access a government website (*Depistage COVID-19*) with a map of open testing centers and their current waiting times (20). The *NHS Covid-19* app, available in England and Wales, offers COVID-19 risk estimates as well. When users enter their postcode, they receive a notification of risk-level (low, medium, high) based on aggregate COVID-19 case information available to local authorities in a given area (21).

A daily symptom checker is integrated into the Irish *COVID Tracker* app, alongside the contact tracing function. This feature enables users to receive personalized recommendations (e.g., self-quarantine, call their physician, request a COVID-19 test) in relation to any symptoms and their severity, and to demographic data voluntarily entered into the system. The French app allows users to connect to a similar symptom checker, hosted on a separate government website (22). The *NHS Covid-19* app, one of the latest to be released in October 2020, integrates a symptom checker tool alongside the option to order a COVID-19 test, via a link to the NHS Test and Trace website. Users can then receive results directly through the app. These new features qualify the app as a medical device, as they enable collection of health data and provide personalized health recommendations to users. The *SwissCovid* and *NHS Covid-19* apps are the only European DCT apps registered as Class I medical devices (23).

The *NHS Covid-19* app also functions as a countdown tool for self-isolation, and a check-in instrument when visiting public venues. The first function, which calculates the length of time a user should self-isolate, activates automatically when a user is notified of contact with a positive case. Based on the encounter date, the app recommends the user self-isolate for 10 days, beginning with the last encounter with the infected person. The countdown tool is also activated when a user enters COVID-19 symptoms or a positive COVID-19 test result into the app. The countdown is initiated, respectively on the day on which symptoms first appear or on the day of the test.

The check-in function allows users to scan a QR code when entering public spaces such as restaurants, bars, shops, cinemas, or religious centers (24, 25). Location data is stored on the user's phone for 21 days. Authorities cannot access this information unless users decide to make it available. NHS documentation explains that the check-in function enables users to record locations visited. App users can then decide to voluntarily disclose this information to contact tracers in case they receive a positive test result. Contact tracers routinely collect information from individuals who test positive (whether they use the NHS app or not) about places visited in the days prior to the test. While individuals have the right not to disclose recent locations that they visited, this information allows contact tracers to alert others who visited the same location. UK health authorities use this information also to assess the level of risk based on the aggregate number of coronavirus cases reported at a particular venue in a certain time period, together with the type of venue (e.g., its architecture). This activity enables health authorities to update the list of places considered to be risky. When public health officials identify a venue as “at risk,” they add to a national reference list that is synchronized with the *NHS Covid-19* app. The app can thus issue an alert to users who have checked in at a risky venue. The tone of the alert message is calibrated according to the level of risk identified by the local health protection team. If risk is high, the user may be urged to call the health authority immediately. The alert does include information about the venue itself.

### Governance and Oversight

Privacy Policies and Terms of Use documents provide information concerning the ethical conditions and legal bases for treatment of personal data by the respective national DCT systems. This information is meant to lend legitimacy to DCT activities, and to reassure the public about legal compliance.

All privacy policy documents of EU member states also make reference to the General Data Protection Regulation (GDPR), particularly concerning the protection of the rights of data subjects ensured by this Europe-wide legislation. For example, the Irish *COVID Tracker* privacy policy declares that “*The app is voluntary to use and the legal basis for the processing of the data is consent—namely Article 6(1)(a) of the GDPR for the processing of personal data and Article 9(2)(a) of the GDPR for the processing of special categories of personal data, in this case health related data*.” (26).

In some cases, data governance principles are also reported. The privacy notice of the Italian DCT app *Immuni* declares compliance with Articles 13-14 of EU GDPR and respect for the principles of privacy (“*Under no circumstances will the users' movements be tracked, thus excluding any form of geolocation*.”), purpose limitation, and data minimization (“*Only the data necessary to alert the users that they have been exposed to a risk of infection, as well as to enable the adoption of any prevention and healthcare measures, are collected*”) (27).

In Switzerland as well, DCT documentation provides the legal basis for the processing of collected data, referencing both existing and new, *ad hoc* provisions: “*The federal legislation on data protection is applicable to the data processing. In addition, the Data Protection Statement is in line with the Epidemics Act of 28 September 2012 (EpG; SR 818.101) and the Ordinance of 24 June 2020 on the Proximity Tracing System for the Coronavirus SARS-CoV-2 (VPTS; SR 818.101.25)”* (28).

These documents frequently mention the role national data protection authorities and various expert bodies played in the early assessment of DCT apps. The FAQ section of *TousAntiCovid*, for example, explains that before the launch of the app, a number of national advisory bodies was consulted on the question of digital tools and privacy protection. The *Conseil Scientifique COVID-19* came out in favor of the app, affirming the usefulness of digital tools in light of the updated “*Test, Alert, Protect*” strategy. Furthermore, the documentation notes the approval of the CNIL (*Commission Nationale Informatique et Libertés*, the French data protection authority), which was responsible for assessing whether adequate data protection measures were in place, both before and after the launch of the app (15).

Documentation from various countries describes the effort to engage a broader array of societal actors in the development of the DCT system. For example, the documentation of the *ProtectScotland* app states that “*The Scottish Government and the NHS Scotland have rigorous information governance process in place. From the early stages of the design of the app, a thorough consultation with relevant Scottish groups of interests and advocacy has taken place, including: The Health and Social Care (Scotland) Public Benet and Privacy Panel; The Scottish Privacy Forum; The Open Rights Group; The COVID-19 Data and Intelligence Network—Data ethics and public engagement subgroup; and representatives of the general public*” (29). However, no details are provided as to public engagement initiatives for the rest of the DCT systems in our sample.

In all cases, the analyzed documentation offers information concerning accountability for the lawful and responsible handling of personal data. For example the German *Corona-Warn-App*'s privacy policy reports that the app “*is provided by the Robert Koch Institute […]. The RKI is also what is called the controller under data protection law, meaning it is responsible for the processing of App users' data. You [the user] can contact the RKI's data protection officer at the above address”*(30). Likewise, the Dutch *CoronaMelder* privacy policy cites the Minister of Health, Welfare and Sport, and the Regional Health Service, as controllers and accountable bodies for the protection of user data against potential abuse, loss, unauthorized access, unwanted disclosures, and unauthorized changes (31). Our study indicates that national governments and departments of health are the authorities responsible for the good functioning of DCT apps, as well as for communication with users and/or intervention when issues arise.

Despite efforts toward the transparent governance of DCT apps, limited information is available about oversight bodies and mechanisms charged with regularly assessing the functioning of DCT systems.

Two exceptions are the commitment by the NHS in England and Wales to review the privacy impact assessment in the event of software updates. As mentioned previously, this app “*is CE marked as Class I medical device in the United Kingdom and developed in compliance with European Commission Directive 93/42/EEC for Class I devices*” (25). As such, the app is subject to stricter oversight regulation (32). The Scottish DCT app also provides some details about the oversight mechanism in place; its documentation states that “*any future changes [to the app] will follow rigorous scrutiny; the decision will be balanced against public health benefit and cost (balanced against other health priorities) and this privacy notice will be updated accordingly for transparency*” (29).

Apart from these two cases, DCT documents do not relay how the responsible institutions intend to monitor an app's activity and the addition of new features over time. Notably, the Dutch documentation stresses that it is the responsibility of the user to check for data information notice updates (which may be introduced with future app developments). These changes will be in immediate effect in the app following publication of the updated privacy policy. Similarly, all of the *Terms of Use* that we analyzed encourage users themselves to inspect the app's source code (via online platforms such as GitHub/GitLab), as well as to report back about their experience of using the app (including any potential issues).

## Discussion

The European approach to DCT has been characterized by marked attention to privacy preservation and data protection. The General Data Protection Regulation (GDPR), in force since May 2018 in European Member States, played a central role in shaping this approach. The GDPR considers the protection of natural persons in relation to personal data processing as a fundamental right (rec. 1 GDPR). Moreover, it recognizes the challenges that new technological developments, together with the global reach of big technology corporations, pose to the protection of personal data (rec. 6 GDPR). Article 25 of the GPDR espouses the principles of data protection by design and by default, making them a legal requirement. These requirements arguably played a key role in shaping the European approach to DCT.

In general, data protection by design asserts that data processing activities should adopt state-of-the-art data protection safeguards across all technical components and processes. Data protection by default refers to the principle that data processing options should automatically be set to the most privacy preserving mode. From a practical point of view, these principles translate into a series of requirements, including data minimization and individual control of personal data. Data minimization contends that only data strictly necessary for a specific purpose should be collected and used, and there must be fixed limits on the extent of processing and the duration of storage and accessibility (art 25.2). Individual control refers to the principle that personal data should be made accessible only upon authorization of data subjects.

These provisions ensure the voluntary nature of European DCT systems, and the selection of privacy-preserving technological solutions for DCT. In particular, the use of GPS-based DCT was never given consideration in Europe, as all countries surveyed recognize Bluetooth-based models as the only legally viable option. In some countries, such as Italy for example, technology experts did not rule out *a priori* the possibility of collecting limited amounts of geolocation data for DCT purposes, but this option never gained support in policy circles. The rationale, based on data protection by design, is that geolocation data is considered redundant to the aim of proximity tracing, since it contains more information than is necessary to notify users about contact with positive cases. However, this argument depends upon a specific view of DCT as a personal warning system, rather than a public health surveillance tool.

European policymakers and advisors however showed a lesser degree of consensus as to the best IT architecture for DCT systems. In the view of some stakeholders, the GDPR did not appear to pose a concrete constraint on specific technological options for DCT. Germany and the UK initially favored a centralized model, to later change to a decentralized one. France and Hungary (not reviewed) ultimately implemented centralized DCT, while remaining fully compliant with GDPR rules. Switzerland, while not a member of the European Union, is revising its Federal Act on Data Protection (FADP) in a way that will also ensure general alignment with the provisions of the GDPR, especially regarding the rights of data subjects. The newly approved law (expected to come into effect in 2022) endorses privacy by design and also by default.

The European model differs in meaningful ways from the DCT approaches adopted by Asian countries in the earliest phases of the pandemic. While it is not possible to speak of an “Asian model” due to the great diversity among DCT systems in Asian countries, it is evident that a more expansive approach characterizes DCT in countries such as China, Hong Kong, Singapore, South Korea, and Taiwan (see Figure 1).

DCT apps developed in China at the beginning of the pandemic became mandatory immediately (33). Hong Kong, Taiwan, and South Korea also deployed mandatory apps and wearable trackers for those living under quarantine, either due to testing positive for COVID-19 or returning from foreign travel (34–36). These apps record GPS geolocation data or use cell tower data to ensure that individuals remain in their homes while in quarantine, and ask the user to enter symptoms, in order to monitor the course of the disease. Taiwan for example used the quarantine DCT feature in combination with rigorous manual contact tracing, which successfully helped contain the spread of the disease (37). South Korea relied on more intrusive surveillance measures, including a number of system tracking citizens' movement, and interactive maps displaying locations visited by COVID-19 positive individuals (38). Singapore was one of the first countries worldwide to introduce a voluntary centralized digital contact tracing app called *TraceTogether*, which was later integrated with a check-in system (called *Safe Entry*) for entry into public spaces (mandatory from the beginning of January 2021) (39). A similar feature was adopted in October 2020 in Hong Kong, where the government is still debating whether the app will be made mandatory.

During the period examined in this study (March to October 2020) European DCT systems showed stability in their overall technical architecture. In all of the reviewed countries, participation in DCT was originally designed to be, and remained, entirely voluntary. Data collection remained limited to randomly generated and periodically deleted Bluetooth IDs. While organizational and technical improvements were implemented to streamline the uploading of positive test results, this process remained fully voluntary, with disclosure of test results possible only with explicit authorization by a DCT user. One partial exception is presented by the England & Wales app, which automatically uploads test results when a test is booked directly through the app.

We have observed an expansion of DCT app features beyond basic proximity tracing in European apps released or updated during the second wave of SARS-CoV-2 that swept through Europe during late summer 2020. Novel features include the capability to track symptoms (Ireland, France, England, and Wales), acquire more detailed epidemiological information about a given area (France, England, and Wales), check in at venues (England and Wales), order COVID-19 tests and access results (England and Wales), and count down the quarantine time (England and Wales).

Our study indicates that privacy preservation through state-of-the-art technological solutions and alignment with data protection laws is the key defining feature of the European approach to DCT. However, we also demonstrated how such an approach is evolving to incorporate novel technological capabilities beyond mere proximity tracing. These developments signal a shift from a strict interpretation of data minimization and purpose limitation, toward a more expansive approach to digital contact tracing in Europe. This evolutionary trajectory seems to reflect technological capacities already seen in Asian countries.

In Europe, the incorporation of novel capacities seems a response to two aims. On one hand, adding features can be viewed as a way to encourage users to download and use DCT apps by offering additional functionalities that users may find useful or interesting. Considering the relatively low level of uptake of DCT apps in European countries compared with the adoption rates needed to ensure effectiveness (40), adding new features may be seen as one way to deliver more personal utility to app users, thus incentivizing participation. On the other hand, novel features such as digital check-ins may increase the aggregate data available to public health authorities, expanding their capacity to monitor how the epidemic is evolving and how the population responds to containment measures. Furthermore, this feature is an ingenious way to integrate manual and digital contact tracing. Both manually and digitally collected information about the whereabouts of positive cases can contribute to map out risky locations. In turn, this information can be used to alert people about potential contacts with positive cases irrespective of whether the use the DCT app or not, thus extending the utility of the DCT app beyond the section of the population that is actually using it.

The panorama of European DCT systems is evolving also in other respects. In December 2020, the privacy policy of the *Corona-Warn-App* was updated, allowing users to record symptoms and retrieve test results (41). In France, the government is considering adding a check-in function to the *TousAntiCovid* app when reopening restaurants (42). These updates may prelude to further expansion of DCT app capabilities in the near future. In Italy for example, the possibility of using the *Immuni* app as a tool in the imminent vaccination campaign is being discussed. The app could evolve into a digital booking system for vaccination appointments, and could then be licensed to store a digital copy of the vaccination certificate for display to health authorities, for entry to designated places or activities (43).

The possible evolution of European DCT systems calls for careful scrutiny and appropriate oversight, especially with respect to GDPR provisions. It must be noted that novel features do not necessarily contravene the principle of data minimization, as they can still be based on minimum necessary data collection for data processing purposes. However, such new features expand the scope of DCT apps beyond the purpose of proximity tracing and warnings to individual users. The legally mandated safeguards regarding data collection and storage may therefore be insufficient to capture additional privacy risks linked to novel functionalities. In other words, data protection by design and by default may be inadequate to address the evolution of DCT systems. To be sure, DCT innovation does not necessarily create greater privacy risks. Such technological evolution should not be prevented, and both public health and ethical rationale support changes aimed at improving the effectiveness of DCT systems against the spread of the virus. Yet as the purpose of DCT apps expands to incorporate new capacities, privacy risks should be regularly reassessed. An adaptive governance approach to DCT seems best suited to regularly fine tuning governance structures and oversight mechanisms over time (44), thus capturing the technical evolution of such systems and their ethical, legal and societal implications.

## Conclusion

As they face subsequent epidemic waves, European countries are tasked with deploying all possible means to mitigate the spread of the virus and minimize the health-related, personal, economic, and social damage it has caused since early 2020. Digital methods offer valuable aid to contain this disaster. In the context of harsh measures and restrictions to individual freedom made necessary by the emergency, DCT is relatively more tolerable, especially in its European incarnation, which offers a comprehensive set of technical and legal safeguards against potential abuse of personal data. Nevertheless, the vast majority of European citizens have not downloaded national DCT apps, despite their ethical and technical robustness. Lack of trust regarding the privacy-preserving features of DCT systems as well as about governments' oversight capacity, may be have contributed to such relatively low figures. Other explanatory hypotheses include insufficient public education campaigns, lack of familiarity on the part of the public with the use of digital health in the context of a public health crisis, and lack of consensus on best practices to implement DCT (14).

In this study we reviewed DCT systems in a number of European countries. We highlighted the strong emphasis that all such systems place on privacy and data protection, their fully voluntary character, and their adoption of the same Bluetooth-based standards for proximity tracing. We noted that such ethical and technological commitment is enshrined in both centralized and decentralized DCT systems. Furthermore, we reported an emerging evolutionary trajectory resulting in the incorporation of novel technological features beyond mere contact tracing that are, to some extent, reminiscent of those already seen in Asia. However, additional policy efforts seem necessary to account for such developments, to gain public trust and to foster more widespread adoption of DCT as a valuable means for containing the effects of the SARS-CoV-2 pandemic.

## Data Availability Statement

The original contributions presented in the study are included in the article/Supplementary Material, further inquiries can be directed to the corresponding author/s.

## Author Contributions

AB and AF contributed to data collection, analysis, manuscript drafting, and editing. EV contributed to writing and editing the manuscript. All authors listed have made a substantial, direct and intellectual contribution to the work, and approved it for publication.

## Conflict of Interest

The authors declare that the research was conducted in the absence of any commercial or financial relationships that could be construed as a potential conflict of interest.

## Footnotes

^1^Translated by the authors.
