# Supplementary material for: Digital Contact Tracing Against COVID-19 in Europe: Current Features and Ongoing Developments
Source: Front Digit Health. 2021 Jun 17;3:660823. doi: 10.3389/fdgth.2021.660823 (PMC8521942; doi:10.3389/fdgth.2021.660823)
Supplement: Supplementary file 1 [file Data_Sheet_1.DOCX]

Supplementary Material

# List of primary documentation (*Privacy Policy*, *Terms of Use, FAQ sections and Press Releases)* of national DCT systems in Europe

| COUNTRY | Primary sources |
| --- | --- |
| Switzerland | - Swiss Federal Office of Public Health. SwissCovid App: Data Protection Statement (2020) [Accessed 31.10.2020]. Available from: <https://web.archive.org/web/20201026031819/https://www.bag.admin.ch/dam/bag/en/dokumente/cc/kom/swisscovid-app-datenschutz.pdf.download.pdf/FOPH_SwissCovid_Data_Protection_Statement_24_June2020.pdf>. - Swiss Federal Office of Public Health. SwissCovid App: Conditions of Use (2020) [Accessed 31.10.2020]. Available from: <https://web.archive.org/web/20210128134805/https://www.bag.admin.ch/dam/bag/en/dokumente/cc/kom/swisscovid-app-nutzungsbedingungen.pdf.download.pdf/FOPH_conditions_of_use_SwissCovid_24_June_2020.pdf> - Swiss Federal Office of Public Health. SwissCovid App: Questions and answers (2020) [Accessed 31.10.2020]. Available from: <https://web.archive.org/web/20200628110526/https://www.bag.admin.ch/dam/bag/en/dokumente/cc/kom/covid-19-faq-tracing-app.pdf.download.pdf/200513_FOPH_FAQ_SwissCovid_App.pdf>. |
| Italy | - Immuni Italia. Immuni: Privacy Notice (2020) [Accessed 31.10.2020]. Available from: <https://web.archive.org/web/20200805213934/https://www.immuni.italia.it/app-pn.html>. - Immuni Italia. Immuni: Termini di utilizzo (2020) [Accessed 31.10.2020]. Available from: <https://web.archive.org/web/20200624215946/https://www.immuni.italia.it/app-tou.html>. - Immuni Italia. Immuni: Do you have any questions? (2020) [Accessed 31.10.2020]. Available from: <https://web.archive.org/web/20201001102027/https://www.immuni.italia.it/faq.html>. |
| Germany | - Robert Koch Institute. Corona-Warn-App: Privacy notice (2020) [Accessed 31.10.2020]. Available from: <https://web.archive.org/web/20201130083701/https://www.coronawarn.app/assets/documents/cwa-privacy-notice-1.3-en.pdf>. - Robert Koch Institute. Corona-Warn-App: Nutzungsbedingungen (2020) [Accessed 31.10.2020]. Available from: <https://web.archive.org/web/20201122065239/https://www.coronawarn.app/assets/documents/cwa-eula-de.pdf>. - Corona-Warn-App. Corona-Warn-App: Frequently Asked Questions (2020) [Accessed 31.10.2020]. Available from: <https://web.archive.org/web/20201031115136/https://www.coronawarn.app/en/faq/#most_asked>. |
| Ireland | - Health Service Executive. COVID Tracker app: Data Protection Information Notice (2020) [Accessed 31.10.2020]. Available from: <https://web.archive.org/web/20201004220235mp_/https://covidtracker.gov.ie/privacy-and-data/data-protection/#6>. - Health Service Executive. COVID Tracker App: Terms of Use (2020) [Accessed 31.10.2020]. Available from: <https://web.archive.org/web/20201031233229if_/https://covidtracker.gov.ie/terms-of-use/>. - Health Service Executive. How the COVID Tracker app works (2020) [Accessed 31.10.2020]. Available from: <https://web.archive.org/web/20201031233229if_/https://covidtracker.gov.ie/how-the-app-works/#close-contact>. |
| Netherlands | - CoronaMelder. CoronaMelder: Privacy Statement (2020) [Accessed 31.10.2020]. Available from: <https://web.archive.org/web/20201015200129/https://coronamelder.nl/en/privacy>. - CoronaMelder. CoronaMelder: Frequently asked questions (2020) [Accessed 31.10.2020]. Available from: <https://web.archive.org/web/20201015200217/https://coronamelder.nl/en/faq>. |
| Scotland | - NHS Scotland. ProtectScotland: Privacy notice (2020) [Accessed 31.10.2020]. Available from: <https://web.archive.org/web/20201030193438/https://protect.scot/privacy-policy-app>. - NHS Scotland. ProtectScotland: How we use your data (2020) [Accessed 31.10.2020]. Available from: <https://web.archive.org/web/20201030192549if_/https://www.protect.scot/how-we-use-your-data>. - NHS Scotland. ProtectScotland: Terms & Conditions (2020) [Accessed 31.10.2020]. Available from: <https://web.archive.org/web/20201030193653if_/https://protect.scot/terms-and-conditions>. - NHS Scotland. ProtectScotland: Frequently asked questions (2020) [Accessed 31.10.2020]. Available from: <https://web.archive.org/web/20201030193108/https://www.protect.scot/faq>. |
| England & Wales | - UK Department of Health and Social Care. NHS COVID-19 app: privacy notice (2020) [Accessed 31.10.2020]. Available from: <https://web.archive.org/web/20201027195058/https://www.gov.uk/government/publications/nhs-covid-19-app-privacy-information/nhs-test-and-trace-app-early-adopter-trial-august-2020-privacy-notice>. - UK Department of Health and Social Care. NHS COVID-19 app: our processing of special categories of personal data (2020) [Accessed 31.10.2020]. Available from: <https://web.archive.org/web/20201016175309/https://www.gov.uk/government/publications/nhs-covid-19-app-privacy-information/nhs-covid-19-app-our-processing-of-special-categories-of-personal-data>. - UK Department of Health and Social Care. NHS COVID-19 app: QR code Check in (2020) [Accessed 31.10.2020]. Available from: <https://web.archive.org/web/20210128162005/https://assets.publishing.service.gov.uk/government/uploads/system/uploads/attachment_data/file/920841/IsolationCountdown_20200923.pdf>. - NHS COVID-19 app support. NHS COVID-19 app: Terms of use (2020) [Accessed 31.10.2020]. Available from: <https://web.archive.org/web/20201030072744/https://covid19.nhs.uk/our-policies.html>. |
| France | - TousAntiCovid. TousAntiCovid: Personal data (2020) [Accessed 31.10.2020]. Available from: <https://web.archive.org/web/20210128151312/https://bonjour.tousanticovid.gouv.fr/privacy-en.html>. - Ministère des solidarités et de la santé. TousAntiCovid: Dossier de presse (2020) [Accessed 31.10.2020]. Available from: <https://web.archive.org/web/20210128152124/https://solidarites-sante.gouv.fr/IMG/pdf/dp_anti-covid_2020.pdf>. - Ministère des solidarités et de la santé. TousAntiCovid: réponses à vos questions (2020) [Accessed 31.10.2020]. Available from: <https://web.archive.org/web/20201031233234/https://solidarites-sante.gouv.fr/soins-et-maladies/maladies/maladies-infectieuses/coronavirus/tousanticovid>. |

# List of secondary sources about national DCT systems in Asia

| COUNTRY | Secondary sources |
| --- | --- |
| China | - BBC News. China launches coronavirus 'close contact detector' app (2020) [Accessed 27.01.2021]. Available from: <https://www.bbc.com/news/technology-51439401>. - CNN Business. China is fighting the coronavirus with a digital QR code. Here's how it works (2020) [Accessed 27.01.2021]. Available from: <https://www.cnn.com/2020/04/15/asia/china-coronavirus-qr-code-intl-hnk/index.html>. - The New York Times. In Coronavirus Fight, China Gives Citizens a Color Code, With Red Flags (2020) [Accessed 27.01.2021]. Available from: <https://www.nytimes.com/2020/03/01/business/china-coronavirus-surveillance.html>. - The Guardian. China's coronavirus health code apps raise concerns over privacy (2020) [updated 2020-04-01Accessed 27.01.2021]. Available from: <http://www.theguardian.com/world/2020/apr/01/chinas-coronavirus-health-code-apps-raise-concerns-over-privacy>. |
| Hong Kong | - Quartz. Hong Kong is using tracker wristbands to geofence people under coronavirus quarantine (2020) [Accessed 27.01.2021]. Available from: <https://qz.com/1822215/hong-kong-uses-tracking-wristbands-for-coronavirus-quarantine/>. - OneZero. I Was Quarantined at Home in Hong Kong and Wore a Tracking Wristband (2020) [Accessed 27.01.2021]. Available from: <https://onezero.medium.com/how-hong-kong-tracks-its-citizens-in-home-quarantine-bab52a7e472f>. - rthk.Hk. Tracing app to launch this month, says CE - RTHK (2020) [Accessed 27.01.2021]. Available from: <https://news.rthk.hk/rthk/en/component/k2/1558046-20201103.htm?spTabChangeable=0>. - LeaveHomeSafe. How It Works? (2021) [Accessed 27.01.2021]. Available from: <https://www.leavehomesafe.gov.hk/en/>. |
| Japan | - Ministry of Health Labour and Welfare. Contact Confirmation Application Privacy Policy (2020) [Accessed 27.01.2021]. Available from: <https://www.mhlw.go.jp/stf/seisakunitsuite/english_pp_00032.html>. - The Verge. Japan rolls out Microsoft-developed COVID-19 contact tracing app (2020) [Accessed 27.01.2021]. Available from: <https://www.theverge.com/2020/6/19/21296603/japan-covid-19-contact-tracking-app-cocoa-released>. - The Japan Times. Japan's new contact-tracing app: 'security blanket' or effective tool? \| The Japan Times (2020) [Accessed 27.01.2021]. Available from: <https://www.japantimes.co.jp/news/2020/05/31/national/japan-contact-tracing-app-privacy-effectiveness/>. |
| Singapore | - TraceTogether. TraceTogether FAQs (2020) [Accessed 27.01.2021]. Available from: <https://support.tracetogether.gov.sg/hc/en-sg/categories/360004509513-TraceTogether-App>. - SafeEntry. National digital check-in system (2020) [Accessed 27.01.2021]. Available from: <https://safeentry.gov.sg/>. - The Straits Times - Singapore. Use of TraceTogether app or token mandatory by end Dec (2020) [Accessed 27.01.2021]. Available from: <https://www.straitstimes.com/singapore/use-of-tracetogether-app-or-token-mandatory-by-end-dec>. - SafeEntry. Places where SafeEntry must be deployed (2021) [Accessed 27.01.2021]. Available from: https://safeentry.gov.sg/. |
| South Korea | - MIT Technology Review. South Korea is watching quarantined citizens with a smartphone app (2020) [Accessed 27.01.2021]. Available from: <https://www.technologyreview.com/2020/03/06/905459/coronavirus-south-korea-smartphone-app-quarantine/>. - The New York Times. Major Security Flaws Found in South Korea Quarantine App (2020) [updated 2020-07-21Accessed 27.01.2021]. Available from: <https://www.nytimes.com/2020/07/21/technology/korea-coronavirus-app-security.html>. - CNN Business. Coronavirus mobile apps are surging in popularity in South Korea (2020) [Accessed 27.01.2021]. Available from: <https://www.cnn.com/2020/02/28/tech/korea-coronavirus-tracking-apps/index.html>. |
| Taiwan | - Quartz. How Taiwan is tracking 55,000 people under home quarantine in real time (2020) [Accessed 27.01.2021]. Available from: <https://qz.com/1825997/taiwan-phone-tracking-system-monitors-55000-under-coronavirus-quarantine/>. - Wired UK. How Taiwan beat Covid-19 (2020) [Accessed 27.01.2021]. Available from: <https://www.wired.co.uk/article/taiwan-coronavirus-covid-response>. - Bloomberg Opinion. If We Must Build a Surveillance State, Let’s Do It Properly (2020) [Accessed 27.01.2021]. Available from: <https://www.bloomberg.com/opinion/articles/2020-04-22/taiwan-offers-the-best-model-for-coronavirus-data-tracking>. |
